# Genetic manipulation of an *Ixodes scapularis* cell line

**DOI:** 10.1128/mbio.02479-23

**Published:** 2024-02-21

**Authors:** Nisha Singh, Agustin Rolandelli, Anya J. O’Neal, L. Rainer Butler, Sourabh Samaddar, Hanna J. Laukaitis-Yousey, Matthew Butnaru, Stephanie E. Mohr, Norbert Perrimon, Joao H. F. Pedra

**Affiliations:** 1 Department of Microbiology and Immunology, School of Medicine, University of Maryland, Baltimore, Maryland, USA; 2 Department of Genetics, Blavatnik Institute, Harvard Medical School, Boston, Massachusetts, USA; 3 Howard Hughes Medical Institute, Chevy Chase, Maryland, USA; Colorado State University, Fort Collins, Colorado, USA

**Keywords:** tick-borne diseases, ticks, rickettsia, Lyme disease, *Borrelia burgdorferi*

## Abstract

Although genetic manipulation is one of the hallmarks of model organisms, its applicability to non-model species has remained difficult due to our limited understanding of their fundamental biology. For instance, manipulation of a cell line originated from the black-legged tick *Ixodes scapularis,* an arthropod that serves as a vector for several human pathogens, has yet to be established. Here, we demonstrate the successful genetic modification of the commonly used tick ISE6 line through ectopic expression and clustered regularly interspaced palindromic repeats [(CRISPR)/CRISPR-associated protein 9 (Cas9)] genome editing. We performed ectopic expression using nucleofection and attained CRISPR-Cas9 editing via homology-dependent recombination. Targeting the E3 ubiquitin ligase x-linked inhibitor of apoptosis (*xiap*) and its substrate *p47* led to an alteration in molecular signaling within the immune deficiency network and increased infection of the rickettsial agent *Anaplasma phagocytophilum* in *I. scapularis* ISE6 cells. Collectively, our findings complement techniques for the genetic engineering of *I. scapularis* ticks*,* which currently limit efficient and scalable molecular genetic screens *in vivo*.

**IMPORTANCE:**

Genetic engineering in arachnids has lagged compared to insects, largely because of substantial differences in their biology. This study unveils the implementation of ectopic expression and CRISPR-Cas9 gene editing in a tick cell line. We introduced fluorescently tagged proteins in ISE6 cells and edited its genome via homology-dependent recombination. We ablated the expression of *xiap* and *p47*, two signaling molecules present in the immune deficiency (IMD) pathway of *Ixodes scapularis*. Impairment of the tick IMD pathway, an analogous network of the tumor necrosis factor receptor in mammals, led to enhanced infection of the rickettsial agent *Anaplasma phagocytophilum*. Altogether, our findings provide a critical technical resource to the scientific community to enable a deeper understanding of biological circuits in the black-legged tick *I. scapularis*.

## OBSERVATION

The black-legged tick *Ixodes scapularis* is a medically relevant chelicerate that transmits several bacteria, viruses, and protozoa to humans and other animals (1, 2). To date, inefficient methods for genetic manipulation in *I. scapularis* make this organism mostly intractable, which leaves significant fundamental gaps in the biology of this ectoparasite. As an example, ectopic expression is a robust tool for elucidating gene function and discovering new phenotypes. However, tick cell lines are reportedly refractory to established transfection methods (3). Additionally, the use of clustered regularly interspaced short palindromic repeats [(CRISPR)/CRISPR-associated protein 9 (Cas9)] (4) remains challenging despite the recent strides made in genome sequencing (5, 6) and the documented *in vivo* application of CRISPR-Cas9 editing to score morphological phenotypes in *I. scapularis* (7). Currently, RNA interference (RNAi) is a widely accepted technique to study functional genomics in ticks (8), but this approach presents limitations, such as off-target effects and transient or low knockdown efficiency (9). Thus, there is a pressing need for the development of genetic tools to manipulate the biology of *I. scapularis* and understand interactions between this arthropod and the microbes it encounters. Here, we report the ectopic expression and CRISPR-Cas9 gene editing of the commonly used ISE6 cell line originated from *I. scapularis*. We indicate the role of the E3 ubiquitin ligase x-linked inhibitor of apoptosis (*xiap*) and *p47* in activating the immune deficiency pathway (10–12). The IMD network is analogous to the tumor necrosis factor receptor pathway in mammals (13, 14) and acts as a primary defense against infection of Gram-negative bacteria in ticks (10–12).

## RESULTS

### Ectopic expression and XIAP-p47 interactions in a tick cell line

**Fig 1:**
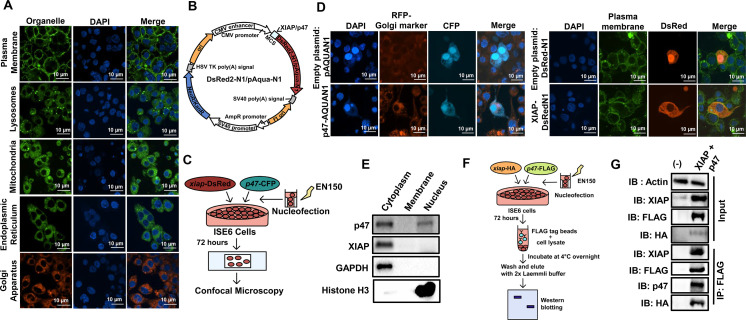
Ectopic expression in the ISE6 cell line. (**A**) Confocal images of ISE6 cells stained with different molecular dyes. (**B**) Cartoon depicting plasmids used for confocal microscopy. *Discosoma* red-N1 (DsRed2-N1) contains the fluorescent gene *DsRed*, whereas p-Aquamarine-N1 (pAqua-N1) carries the fluorescent gene *Aqua*. (**C**) Schematic representation of ectopic expression in tick cells. (**D**) Ectopic expression of Aqua-tagged p47 and DsRed-tagged XIAP in ISE6 cells. ISE6 cells were nucleofected with the plasmid containing *p47-Aqua*, *xiap-DsRed*, or the empty vector (DsRed-N1 or pAqua-N1); Golgi (red), plasma membrane (green), and DAPI (blue). (**E**) Sub-cellular fractionation of ISE6 cells. Glyceraldehyde 3-phosphate dehydrogenase (GAPDH) and histone H3 were used as cytosolic and nuclear markers, respectively. (**F**) Schematic representation of pull-down in ISE6 cells. *xiap*-HA and *p47*-FLAG constructs were nucleofected in ISE6 cells (3 × 10^7^). Co-transfected cells were harvested after 72 hours, and 10 mg of the lysate was incubated with 50 µL of FLAG beads for 18 hours at 4°C. (**G**) The complex was immunoprecipitated (IP) using the 3X-FLAG peptide and subjected to immunoblotting (IB). Data represent one of two independent experiments.

To determine whether fluorescent probes might be used to visualize subcellular structures in *I. scapularis*, we stained organelles within ISE6 cells through commonly used molecular dyes for the plasma membrane, lysosome, mitochondria, endoplasmic reticulum, and Golgi apparatus (Fig. 1A). We then characterized two previously identified proteins from *I. scapularis*: XIAP and p47 (10–12). p47 is an enzymatic substrate of the E3 ubiquitin ligase XIAP and activates the tick IMD pathway through Kenny (also known as IKKγ/NEMO) in response to infection (10). The impairment of *p47* expression through RNAi in *I. scapularis* reduces Kenny accumulation, lessens phosphorylation of IKKβ (IRD5), and diminishes cleavage of the nuclear factor-κB molecule Relish in *I. scapularis* (10).

We cloned *p47* into the pAquamarine-N1 (*p47*-Aqua) plasmid and *xiap* in the *Discosoma* red-N1 (XIAP-DsRed) plasmid (Fig. 1B). We successfully developed a protocol to nucleofect these plasmids into tick cells (Fig. 1C). Confocal microscopy revealed that the recombinant protein p47-Aqua localized in the nucleus and the cytosol, whereas XIAP-DsRed was predominantly detected in the cytosol of ISE6 cells (Fig. 1D). Sub-cellular fractionation of nucleofected cells independently confirmed the location of p47 and XIAP in ISE6 cells (Fig. 1E). McClure et al. (10) demonstrated that XIAP binds and ubiquitinylates p47 in a lysine (K)-63 dependent manner in human embryonic kidney 293T cells. To take advantage of the developed protocol for ectopic expression in tick cells, we next performed co-immunoprecipitation using the XIAP-HA and p47-FLAG vectors (Fig. 1F). We detected molecular interactions between XIAP and p47 via affinity purification in ISE6 cells (Fig. 1G). These data demonstrate the ectopic expression of immune molecules in ISE6 cells and confirm protein-protein interactions in *I. scapularis*.

### CRISPR-Cas9 genome editing in the ISE6 cell line

**Fig 2:**
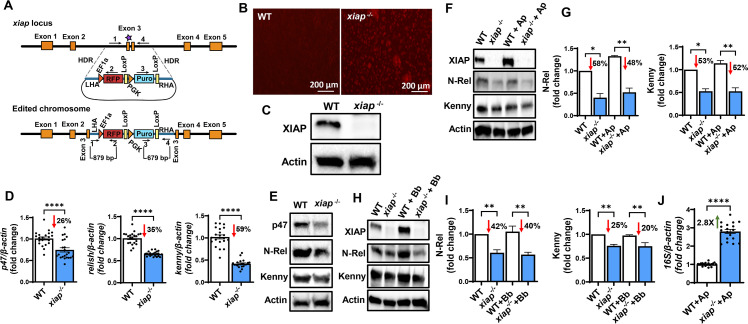
CRISPR-Cas9 *xiap* editing in ISE6 cells. (**A**) Schematic representation of the *xiap* locus and the donor construct. The orange boxes represent the five exons of *xiap* with sgRNA binding and the Cas9 cleavage site on exon 3 (purple star). The donor construct contains the red fluorescent protein (RFP) and puromycin cassette (RFP-Puro) along with the DNA fragments of ~600 bp in length, homologous to the *xiap* locus, flanking the Cas9 cleavage site on the 5′ and 3′ ends for HDR. The arrows with numbers 1–4 represent the primers for the amplification analysis. (**B and C**) Editing was confirmed via fluorescence microscopy (**B**) and western blotting (**C**). (**D and E**) Functional disruption of *xiap* impaired the expression of molecules associated with the IMD pathway at both transcriptional (**D**) and translational levels (**E**). (**F–I**) 3 × 10^6^ wild-type (WT) and *xiap^-/-^* cells were plated in a 6-well plate and stimulated with *Anaplasma phagocytophilum* [multiplicity of infection (MOI) 50] or *Borrelia burgdorferi* (MOI 50) for 15 minutes. Disruption of XIAP signaling impairs Kenny accumulation and Relish cleavage in response to (**F and G**) *A. phagocytophilum* infection or (**H and I**) *B. burgdorferi* stimulation. For data normalization, Kenny and N-Rel band densities were normalized to actin, and values were divided by the uninfected WT control densitometry. Western blot images are a representative image of at least two independent experiments. (**J**) WT and *xiap^-/-^* cells were infected with *A. phagocytophilum* (MOI 50). ISE6 cells were harvested after 72 hours of infection. The *A. phagocytophilum* 16S rRNA transcript was quantified by qRT-PCR, and the expression data were normalized to *I. scapularis* β-actin. The qRT-PCR data show the combination of three independent experiments. Results are represented as a mean ± SEM. Statistical significance was evaluated by unpaired *t* test with Welch’s correction (**D and J**) and one-way ANOVA with *post hoc* Tukey test (**G and I**). *P < 0.05; **P < 0.01; and ****P < 0.0001.

We performed *xiap* and *p47* genome editing via CRISPR-Cas9 in the ISE6 cell line. We isolated genomic DNA from ISE6 cells and designed PCR primers to amplify 1,000 base pairs (bp) of the target regions. Specifically, two single guide RNAs (sgRNAs) per gene were made for *xiap* and *p47* (Tables S1 and S2). We developed a methodology for CRISPR-Cas9 through homology-directed repair (HDR) (Fig. 2; Fig. S1–S4). Genome manipulation via HDR displays a precise editing mechanism when a template is introduced into cells as a donor for homologous recombination (15). We used a cassette with an antibiotic marker and a reporter gene, along with DNA fragments homologous to the target gene. These homologous regions were positioned on either side of the donor cassette to facilitate recombination (Fig. 2A; Fig. S3A). Due to the low efficiency associated with transfecting tick lines, we employed a ribonucleoprotein (RNP) delivery method to introduce the Cas9 endonuclease protein and sgRNAs in ISE6 cells (Fig. S1). Following nucleofection, cells were split and selected with puromycin (Fig. S2). We chose antibiotic selection over cell sorting because it remains technically unfeasible to culture tick cells from a single clone.

For validation of the editing event, a primer set was designed to target both *p47-* and *xiap*-edited cells amplifying the left homology arm and the red fluorescent protein gene. A second primer pair was engineered to cover the puromycin cassette and the right homology arm (Fig. 2A; Fig. S3A). PCR amplification detected the donor cassette insertion in the edited *p47^-/-^* and *xiap^-/-^* ISE6 cells (Fig. S3B and S4A). Knock-in events were orthogonally confirmed through Sanger sequencing (Fig. S3C and S4B) and fluorescence microscopy (Fig. 2B and S3D). Western blot revealed the absence of the wild-type (WT) protein bands for p47 and XIAP in edited ISE6 cells (Fig. S3E and 2C). Disruption of a *p47* homolog in the budding yeast *Saccharomyces cerevisiae*, named Shp1, leads to lethality (16). Similarly, edited *p47^-/-^* cells did not survive the puromycin selection procedure, likely due to the involvement of *p47* in growth. These findings confirmed the delivery of Cas9 RNPs and independent ablation of both *p47* and *xiap* in ISE6 cells.

### Functional disruption of *xiap* impairs IMD signaling pathway in *I. scapularis*

We then asked whether cells deficient in *xiap* (*xiap*^-/-^) were impaired for the IMD signaling pathway (Fig. S5) (10–12). Editing of *xiap* affected the transcription and translation of *p47*, *relish,* and *kenny* (IKKγ/NEMO) and the cleavage of the NF-κB molecule Relish in ISE6 cells (Fig. 2D and E). We determined the effect of *xiap* editing in ISE6 cells during microbial stimulation with the Lyme disease spirochete *Borrelia burgdorferi* or the rickettsial agent *Anaplasma phagocytophilum*. A significant decrease in the accumulation of Kenny and reduced nuclear translocation of N-Rel were observed in *xiap*^-/-^ cells during microbial stimulation (Fig. 2F through I), indicating that functional disruption of *xiap* results in the impairment of the IMD pathway in ticks. Importantly, WT and *xiap^-/-^* ISE6 cells were infected with *A. phagocytophilum* for 72 hours, and bacterial burden was assessed by RT-qPCR. We observed an increase in *A. phagocytophilum* load in *xiap^-/-^* ISE6 cells compared to the control treatment (Fig. 2J). These data obtained through CRISPR editing implicated the E3 ubiquitin ligase XIAP as an important molecule for the *I. scapularis* IMD pathway.

## DISCUSSION

Genetic manipulation of ticks lags behind insects and our study expands the genetic toolbox in *I. scapularis*. We (i) expressed two tick proteins in ISE6 cells, XIAP and p47 (10–12); (ii) confirmed their previous molecular interactions (10); and (iii) successfully detected their subcellular location. We also developed a protocol for CRISPR-Cas9 gene editing in ISE6 cells and provided evidence that disrupting components of the IMD pathway resulted in an increase in *A. phagocytophilum* burden in *I. scapularis*. Our approach follows an earlier report in which Kurtti et al. (17) used cationic lipid-based transfection reagents to deliver a red fluorescent protein and a selectable marker, neomycin phosphotransferase, into ISE6 cells.

Recent advancements used embryo injection of CRISPR-Cas9 through the Receptor-Mediated Ovary Transduction of Cargo (ReMOT Control) (18) for direct delivery of the Cas9-RNP complex into *I. scapularis* (7). Although this technology is a breakthrough for the tick community, there are significant limitations for its applicability *in vivo*, including low survival and efficiency in addition to the long life cycle of *I. scapularis* ticks. Our study provides a complementary approach and paves the way for exploring ancillary CRISPR-Cas9 technologies, including CRISPR activation (19) and CRISPR interference (20). The development of genetic tools for tick research offers unique avenues to identify crucial genes related to physiology, immune signaling, and the detection of microbes. Ectopic expression and CRISPR technologies in cells might aid in the identification of antigen targets for the development of tick-based vaccines (21), epitopes associated with the α-gal allergy to red meat (22), and proteins linked to acaracide resistance (23).

## SUPPLEMENTAL MATERIAL

Supplemental Material mbio.02479-23-s0001.pdf Supplemental text, figures, and tables.

ASM does not own the copyrights to Supplemental Material that may be linked to, or accessed through, an article. The authors have granted ASM a non-exclusive, world-wide license to publish the Supplemental Material files. Please contact the corresponding author directly for reuse.
