# Supplementary material for: Genetic manipulation of an Ixodes scapularis cell line
Source: mBio. 2024 Feb 21;15(3):e02479-23. doi: 10.1128/mbio.02479-23 (PMC10936194; doi:10.1128/mbio.02479-23)
Supplement: Supplemental Material — Supplemental text, figures, and tables. [file mbio.02479-23-s0001.pdf]

Supplementary Information

Materials and Methods

Supplementary Figures and Legends

Figures S1 to S5; Tables S1 to S2

Genetic manipulation of an *Ixodes scapularis* cell line

Authors: Nisha Singh[1α], Agustin Rolandelli[1], Anya J. O'Neal[1*], L. Rainer Butler[1†], Sourabh Samaddar[1], Hanna J. Laukaitis-Yousey[1], Matthew Butnaru[2,3], Stephanie E. Mohr[2], Norbert Perrimon[2, 3] and Joao H. F. Pedra[1#]

Affiliations: [1]Department of Microbiology and Immunology, University of Maryland, School of Medicine, Baltimore, MD 21201, USA

[2]Department of Genetics, Blavatnik Institute, Harvard Medical School, Boston, MA 02115, USA

[3]Howard Hughes Medical Institute, Chevy Chase, MD, 20815, USA

[α]Present address: Department of Biotechnology, School of Energy Technology, Pandit Deendayal Energy University Knowledge Corridor, Gandhinagar, Gujarat 382426, India

[*]Present address: Immunology Program, Memorial Sloan Kettering Cancer Center, New York, NY 10065, USA

[†]Present address: Department of Genetics, Blavatnik Institute, Harvard Medical School, Boston, MA 02115, USA

[#]Corresponding Author: jpedra@som.umaryland.edu

Materials and Methods

Tick cell culture

All experiments were performed under guidelines from the NIH and approved by the Institutional Biosafety Committee (IBC-00002247) at the University of Maryland, Baltimore. The embryonic cell line (ISE6) was cultured at 34°C in L15C300 medium supplemented with 10% heat inactivated fetal bovine serum (FBS, Millipore Sigma), 0.1% bovine lipoprotein concentrate (LPPC, MP Biomedicals) and 10% tryptose phosphate broth (TPP, BD). ISE6 cells were grown to confluency in T25 flasks (Greiner) and verified by PCR to be *Mycoplasma* free (Southern Biotech).

Plasmids

xiap and *p47* were cloned in the pCMV-HA and pCMV-FLAG vectors, respectively, as previously described (1). For confocal microscopy, *xiap* and *p47* were cloned in DsRed2-N1(a gift from Michael Davidson, Addgene plasmid #54493) and pAQUA-N1vectors (a gift from Fabienne Merola, Addgene plasmid #42888). Genes of interest were amplified from ISE6 cells complementary DNA (cDNA) using Phusion polymerase (NEB Biolabs). *xiap* and *p47* were cloned between the restriction sites *SacI*/*EcoRI* and *SalI*/*BamHI*, respectively. The *xiap*- and *p47*- donor DNA used in CRISPR experiments were procured from Origene technologies with a customized RFP-Puro cassette. All constructs were verified through Sanger sequencing.

Bacteria

Escherichia coli BL21 (DE3) was cultured overnight at 37°C in lysogeny broth (LB) supplemented with 100 μg/ml ampicillin. *A. phagocytophilum* strain HZ was cultured in the human leukemia cell line, HL-60 cell line (ATCC, CCL-240) (2). Host-free *A. phagocytophilum* was obtained by collecting the infected-HL-60 cells at 3,260 x g for 10 minutes. The pellet was resuspended in L15C300 medium and lysed by passing through a 27 ½ gauge needle five

times. Cell debris was separated by centrifugation at 750 x g for 5 minutes at 4°C. Host-free bacteria were enumerated using the following formula: number of infected HL-60 cells × 5 morulae/cell × 19 bacteria/morulae × 0.5 recovery rate (3). Low passage *B. burgdorferi* B31 clone MSK5 was cultured in Barbour-Stoenner-Kelly (BSK)-II medium supplemented with 6% normal rabbit serum at 37°C, as previously described (4, 5). The *B. burgdorferi* genome consists of 21 linear and circular plasmids, which are important for infectivity in mice. Total DNA was extracted from a liquid culture and plasmid profiling was performed by PCR amplification of necessary virulence plasmids using REDTaq DNA polymerase (4).

Antibody generation

The *I. scapularis* Kenny custom antibody used in this study was generated by Genscript. Rabbits were immunized three times with 0.2 mg of the tick Kenny immunogen (amino acids 223-356) for antibody generation. The *I. scapularis* Relish monoclonal custom antibody used in this study was generated by Genscript. Mice were immunized three times with 0.2 mg of the tick N-Rel immunogen (Rel homology domain; amino acids 19-192).

Nucleofection

For nucleofection, 2 x 10^5 ISE6 cells were pelleted by centrifugation at 100 x g for 10 minutes. The pellet was washed with 10 ml of 1 x PBS and resuspended in 20 µl of nucleofection SF buffer (Lonza Biosciences), in which 600 ng of the DsRed2-N1 plasmid was added to the suspension. The nucleofection mix was transferred to a multi-well cuvette and subjected to the EN150 pulse condition using a 4D-Nucleofector system (Lonza Biosciences). Following nucleofection, cells were incubated in the cuvette for 10 minutes at room temperature. ISE6 cells were added to pre-warmed L15C complete media in a 12-well plate and observed for fluorescence by microscopy after 72 hours (6).

Pull-down assays

Following nucleofection of 4 x 10^8 ISE6 cells, the pellet was resuspended in 800 µl of SF buffer. Twenty-five µg of either *p47*-FLAG or *xiap*-HA plasmids was added to the cell suspension. The cell suspension was split among eight nucleofection cuvettes (100 µl each) and pulsed using EN150. After 10 minutes post-nucleofection, tick cells were added to a T25 flask containing 5 ml of pre-warmed L15C complete medium and incubated at 34 °C with 1% CO_2 for 72 hours. Cells were collected, washed twice with 1 x PBS, and lysed in immunoprecipitation lysis buffer (Thermo Scientific). The lysate (10 mg) was incubated with 300 µl of anti-FLAG cross-linked agarose beads overnight at 4°C. Beads were washed three times with 200 mM NaCl added to 1 x PBS and boiled for 5 minutes in 2X Laemmli buffer to elute proteins. Eluted proteins and input samples were analyzed by western blot for XIAP-HA and p47-FLAG detection.

Confocal microscopy

For microscopy, 5 x 10^5 ISE6 cells were nucleofected with 500 ng of plasmid (*xiap*-DsRed-N1 or *p47*-AQUA-N1). Tick cells were then plated on a glass coverslip (Corning). After 72 hours, cells were stained with the molecular dyes (Supplementary Table 1), as per the manufacturer's protocol. ISE6 cells were fixed with 4% paraformaldehyde followed by 1 x PBS washes and the coverslip was mounted on a slide using Antifade gold mounting reagent with 4′,6-diamidino-2-phenylindole (DAPI) (Invitrogen) and observed under the Nikon W-1 Spinning Disk confocal microscope. The following laser channels were used: 561 nm (XIAP-DsRed), 488 nm (GFP, plasma membrane, lysosomes, mitochondria, and endoplasmic reticulum), 405 nm (DAPI), 456 nm (p47-AQUA) and 561 nm (RFP, Golgi apparatus).

Subcellular fractionation

Subcellular fractionation was performed as previously described (7). Briefly, ISE6 cells were resuspended in 500 µl fractionation buffer and passed through a 27-gauge needle 10 times. After a 20-minute incubation on ice, the cells were centrifuged at 720 x g for 5 minutes. The pellet comprised of nuclei and the supernatant contained cytoplasm and membranes. The nuclear pellet was resuspended in 500 µl fractionation buffer and passed through a 25-gauge needle 10 times. The sample was then centrifuged at 720 x g for 10 minutes and resuspended in TBS with 0.1% Sodium dodecyl sulfate (SDS). The fraction containing cytoplasm and membrane components was centrifuged at 10,000 x g for 5 minutes and the supernatant was collected. The supernatant was then ultra-centrifuged at 100,000 x g for 1 hour. The pellet was washed in 400 µl of fractionation buffer, passed through 25-gauge needle and re-centrifuged at 100,000 x g for 45 minutes. The membrane pellet was resuspended in TBS containing 0.1 % Sodium dodecyl sulfate (SDS) for western blot detection.

sgRNA and donor DNA design

Amplicons were validated using Sanger sequencing and aligned to the reference genome. Exons were identified using the ORF finder tool (https://www.bioinformatics.org/sms2/orf_find.html). Exon 3 for *xiap* and exon 5 for *p47* were selected. The CHOPCHOP server (https://chopchop.cbu.uib.no/) was used to identify guide RNA hits. The sgRNAs were selected based on the ~20 bp sequences adjacent to NGG-PAM (protospacer adjacent motif) with 40-80% GC content and no off-target binding. The sgRNA scaffold contained the CRISPR RNA (crRNA) or the ~20 bp target sequence, as well as the transactivating CRISPR RNA (tracrRNA) (8, 9). Two sgRNAs sequences were selected per gene, one targeting each strand (Supplementary Table 2) and customized from Synthego. The *sp*Cas9 protein and sgRNAs were combined *in vitro* to form the RNP complex which was then introduced into the ISE6 cells through nucleofection, along with the donor DNA.

To induce HDR, a donor DNA or DNA repair template was delivered to cells along with the sgRNA and Cas9 endonuclease (9-11). The donor DNA constructs targeting *xiap* and *p47* genes had the following features: (*i*) a red fluorescent protein (RFP) driven by the human elongation factor 1 α (EF1α) promoter; (*ii*) the puromycin gene for antibiotic based selection flanked by a phosphoglycerate kinase 1 (PGK) promoter; (*iii*) the loxp (locus of X-over P1) sites to flox out the puromycin cassette; and (*iv*) DNA fragments of ~600 bp in length, homologous to the *xiap* or *p47* gene locus flanking the Cas9 cleavage site on the 5' and 3' ends. The resulting plasmid was of ~7 Kb in length with the 2.5 Kb RFP-Puro cassette targeted for insertion at the *xiap* or the *p47* gene loci.

CRISPR-Cas9 gene editing in tick cells

To prepare the RNP, 200 pmol of Cas9-NLS-tagRFP protein (Genaxxon bioscience) and 100 µM of sgRNAs for *p47* and *xiap* (Supplementary Tables 1-2) were mixed and incubated at room temperature for 20 minutes. The RNP complexed together with 7 µg of donor DNA was nucleofected into 3 X 10^7 ISE6 cells using the EN150 pulse code and buffer SF via the 4D-Nucleofector system (Lonza Bioscience) (Figure S1). 3 days post-nucleofection, ISE6 cells were split at a 1:10 ratio and cultured for 10 days followed by another round of splitting. This process was repeated for 7 cycles (75 days) to eliminate any extraneous effect of the donor DNA. The edited cells were selected using puromycin (4 µg/ml). The dose of puromycin was determined with a kill curve and media with fresh antibiotic was changed every other day. After puromycin selection, ISE6-edited cells were cultured without antibiotic to promote population expansion. Finally, ISE6-edited cells were maintained in ISE6 cell media with antibiotic at the concentration of 0.4 µg/ml.

Antibiotic kill curve

5×10^5 ISE6 cells/well were seeded in a 12-well plate and incubated overnight at 34°C. After 24 hours, the medium was replaced with varying concentrations of puromycin ranging from 0 - 10 µg/ml. Each concentration was maintained in triplicate. The medium containing puromycin was replaced every two days for a duration of 10 days. Measurement of cell viability through trypan blue determined the effect of puromycin on ISE6 cells and the kill curve was plotted (Figure S2). The lowest concentration of puromycin that completely blocked the growth of tick cells was used for the selection process.

Quantitative reverse transcription polymerase chain reaction (qRT-PCR)

RNA was extracted from the cells preserved in TRIzol (Invitrogen) using the PureLink RNA Mini kit (Invitrogen) and the cDNA was synthesized with the Verso cDNA Synthesis Kit using 500-800 ng of RNA (Thermo Scientific). For quantification of gene expression through qRT-PCR, 2X iTaq™ Universal SYBR® Green Supermix from Bio-rad was used. To measure gene expression, 1 µl of cDNA along with SYBR green was used and quantified using a CFX96 Touch Real-Time PCR Detection System (Bio-rad). All target genes were amplified at 54°C, except for *A. phagocytophilum* 16S, which was amplified at 48°C. The qRT-PCR conditions used were: (1) 95°C for 3 minutes; (2) 33 cycles of 95°C for 10 seconds, 48 or 54°C for 30 seconds; and the (3) melting curve analysis to confirm the specificity of the reaction. To ensure the absence of primer-dimer formation and/or contamination, no-template controls were incorporated into the assay. Each sample and control reactions were conducted in duplicate. Gene expression was measured using the primers listed in Supplementary Table 2.

Western blotting

ISE6 cells (3×10^6 cells per well) were plated in 6-well plates (Millipore Sigma) and stimulated accordingly. Protein lysate was prepared in Radio-immunoprecipitation assay (RIPA)

buffer (Merck Millipore) with a protease inhibitor cocktail (Roche) and protein concentration was estimated using BCA assay (Thermo Scientific). Sodium dodecyl sulfate polyacrylamide gel electrophoresis (SDS-PAGE) samples were prepared by boiling equal amounts of protein in 6X Laemmli sample buffer (Alfa Aesar) containing 5% β-mercaptoethanol. Proteins were transferred onto PVDF membranes (Biorad) and membrane was blocked for 1 hour with 5% skimmed milk prepared in PBS-T. Primary antibodies were incubated overnight at 4°C in PBS-T and blots were washed four times in PBS-T. Blots were subsequently incubated with secondary antibodies for at least 1 hour at room temperature with gentle rocking. Blots were washed four times in PBS-T, incubated with enhanced chemiluminescence (ECL) substrate solution for 1 minute (Millipore), and imaged.

Statistical Analysis

Three independent experiments were performed for each set of experiments. Means ± standard error of the mean (SEM) was plotted, and statistical significance was assessed by the unpaired *t* test with Welch's correction or one-way analysis of variance (ANOVA). GraphPad PRISM® (GraphPad Software version 9.1.0) was used for statistical analyses and GraphPad Quickcals program was used for outlier detection (https://www.graphpad.com/quickcalcs/Grubbs1.cfm). A p value<0.05 was considered statistically significant.

Supplementary Figures

Figure S1. Schematic of the ribonucleoprotein (RNP) delivery and selection in tick cells. 200 pmol of the Cas9-NLS-tag RFP protein (Cas9 endonuclease tagged with nuclear localization sequence and red fluorescent protein) and 100 μM of sgRNA 1 and 2 were mixed and incubated at room temperature to prepare the RNPs. The RNP complex together with 7 μg of the donor DNA was nucleofected in 3 x 10^7 ISE6 cells using the program code EN150 and the buffer SF on the 4D-Nucleofector System. ISE6 cells were then passaged for 7 cycles followed by puromycin selection, genomic DNA isolation and gene amplification analysis.

Figure S2. Puromycin kill curve in tick cells. 0.5 x 10^6 ISE6 cells were seeded in a 12-well plate. After 24 hours, fresh L15C300 (Leibovitz-15 media) media with puromycin (0-10 μg/ml) was added to the cells. ISE6 cells were replenished with fresh media and antibiotic every 48 hours for 10 days. The viability of ISE6 cells was estimated using trypan blue.

Figure S3. CRISPR-Cas9 *p47* editing in ISE6 cells. (A) Schematic representation of the *p47* locus and the donor construct. The orange boxes represent the eight exons of the *p47* locus, and the black line represents the intron sequence (top). The purple star on exon 5 represents the sgRNA binding and Cas9 cleavage site. The donor DNA (bottom) carries the promoter and coding sequence for the red fluorescent protein (RFP)-puromycin cassette (RFP-Puro) with the loxp (locus of X-over P1) sites in the pUC19 backbone. The donor DNA construct also carries DNA fragments of ~600 bp in length, homologous to the *p47* gene locus, flanking the Cas9 cleavage site on the 5’ and 3’ ends for homology-directed repair (HDR). The arrows with numbers 1-4 represent primers for gene amplification analysis. (B) PCR amplification confirming the integration of the donor cassette (RFP-Puro) in the genomic DNA prepared from *p47* edited ($p47^{-/-}$) cells. PCR was performed using the primer pairs 1 and 2 and 3 and 4, as mentioned in (A). The genomic DNA prepared from wildtype (WT) ISE6 cells did not show any amplification.

The donor DNA was used as a positive control (+). The length in nucleotides of the amplified fragments is represented in numbers. (C) Editing was further confirmed through Sanger sequencing where the blue arrow is the targeted exon (Exon 5), red and dark blue arrows represent the sgRNA target sites with arrowheads showing the binding direction. The number in parenthesis is the binding location of sgRNA on the exon. The results were validated through fluorescence microscopy (D) and western blotting (E).

Figure S4. Confirmation of *xiap* editing in ISE6 cells. (A) PCR amplification confirming the integration of the donor cassette (RFP-Puro) in the genomic DNA prepared from *xiap* edited (*xiap*$^{-/-}$) cells. PCR was performed using either the primer pairs 1 and 2 or the primer set 3 and 4, as mentioned in Figure 2A. The genomic DNA prepared from wildtype (WT) ISE6 cells did not show any amplification. The donor DNA was used as a positive control (+). The length in nucleotides of the amplified fragments is represented in numbers. (B) Editing was further confirmed through Sanger sequencing where the blue arrow is the targeted exon (Exon 3), red and dark blue arrows represent the sgRNA target sites with arrowheads showing the binding direction. The number in parenthesis is the binding location of sgRNA on the exon.

Figure S5. The IMD pathway in ticks. The binding of the infection-derived lipid POPG to Croquemort (Crq), relays a signal to TAK1 through an unknown mechanism. This cascade of events results in the polyubiquitylation of p47 in a K63-dependent manner by the E3 ubiquitin ligase XIAP leading to the cleavage and translocation of Relish into the nucleus. The transcription factor Relish promotes expression of the antimicrobial peptide ctenidin-1, enhancing the tick antibacterial response.

References

1. McClure Carroll EE, Wang X, Shaw DK, O'Neal AJ, Oliva Chavez AS, Brown LJ, Boradia VM, Hammond HL, Pedra JHF. 2019. p47 licenses activation of the immune deficiency pathway in the tick *Ixodes scapularis*. Proc Natl Acad Sci U S A 116:205-210.
2. Carlyon JA. 2005. Laboratory maintenance of *Anaplasma phagocytophilum*. Curr Protoc Microbiol Chapter 3:Unit 3A 2.
3. Yoshiie K, Kim HY, Mott J, Rikihisa Y. 2000. Intracellular infection by the human granulocytic ehrlichiosis agent inhibits human neutrophil apoptosis. Infect Immun 68:1125-33.
4. Labandeira-Rey M, Skare JT. 2001. Decreased infectivity in *Borrelia burgdorferi* strain B31 is associated with loss of linear plasmid 25 or 28-1. Infect Immun 69:446-55.
5. Zuckert WR. 2007. Laboratory maintenance of *Borrelia burgdorferi*. Curr Protoc Microbiol Chapter 12:Unit 12C 1.
6. O'Neal AJ, Singh N, Rolandelli A, Laukaitis HJ, Wang X, Shaw DK, Young BD, Narasimhan S, Dutta S, Snyder GA, Samaddar S, Marnin L, Butler LR, Mendes MT, Cabrera Paz FE, Valencia LM, Sundberg EJ, Fikrig E, Pal U, Weber DJ, Pedra JHF. 2023. Croquemort elicits activation of the immune deficiency pathway in ticks. Proc Natl Acad Sci U S A 120:e2208673120.
7. Abcam. 2022. Subcellular fractionation protocol.
8. Gasiunas G, Barrangou R, Horvath P, Siksnys V. 2012. Cas9-crRNA ribonucleoprotein complex mediates specific DNA cleavage for adaptive immunity in bacteria. Proc Natl Acad Sci U S A 109:E2579-86.
9. Ran FA, Hsu PD, Wright J, Agarwala V, Scott DA, Zhang F. 2013. Genome engineering using the CRISPR-Cas9 system. Nat Protoc 8:2281-2308.

10. Yang L, Yang JL, Byrne S, Pan J, Church GM. 2014. CRISPR/Cas9-directed genome editing of cultured cells. Curr Protoc Mol Biol 107:31 1 1-17.

11. Gantz VM, Bier E. 2015. Genome editing. The mutagenic chain reaction: a method for converting heterozygous to homozygous mutations. Science 348:442-4.

Supplementary Table 1: Resources and reagents available

Antibody	Source	Identifier	Dilution/Concentration
Mouse Anti-*I. scapularis* Relish monoclonal Ab	GenScript	custom	1:500
Histone H3 Rabbit polyclonal Ab	Cell Signaling	9715	1:1,000
GAPDH (14C10) Rabbit mAb	Cell Signaling	2118S	1:1,000
Goat Anti-Rabbit IgG H&L (HRP)	Abcam	ab97051	1:4,000-10,000
Rabbit Anti-Mouse Actin	Millipore Sigma	A2103	1:4,000
HA-Tag (C29F4) Rabbit mAb	Cell Signaling	3724	1:1,000
Anti-Flag	Millipore Sigma	F3165	1:1,000
Anti-NSFL1C mouse monoclonal Ab	Abnova	H00055968-B01P	1:500
Rat monoclonal [H139-52.1] Anti-Mouse kappa light chain (HRP)	Abcam	ab99632; discontinued	1:4,000
Rabbit Anti-*I. scapularis* XIAP polyclonal Ab	Thermo Scientific	custom	1:1,000
Rabbit Anti-*I. scapularis* Kenny polyclonal Ab	GenScript	custom	1: 1,000
Goat Anti-mouse IgG H+L(HRP)	Elabscience	E-AB-1001	1:3,000
**Cell Dye**			
CellBrite^{TM} Fix membrane stain	Biotium	30090	1:1,000
Hoechst 33342 Nuclear Stain	AAT Bioquest	17533	1:1,000
SlowFade Gold Antifade mountant	Invitrogen	S36938	N/A
CytoPainter LysoGreen indicator reagent	Abcam	ab176826	1:1,000
BioTracker^{TM} 488 Green Mitochondria dye	Millipore	SCT136	1:1,000
Cell Navigator^{TM} TMR Ceramide Golgi staining kit- red fluorescence	AAT Bioquest	22752	1:1,000

CytoPainter ER staining kit - green fluorescence	Abcam	ab139481	1:1,000
**Restriction Enzymes**			
SacI-HF®	New England BioLabs	R3156S	N/A
EcoRI-HF®	New England BioLabs	R3101S	N/A
BamHI-HF®	New England BioLabs	R3136S	N/A
SalI-HF®	New England BioLabs	R3138S	N/A
**Cell Media**			
Leibovitz's L-15 Medium, powder	Gibco	41300039	N/A
L-aspartic acid	Millipore-Sigma	11189	3.37 mM
L-glutamine	Millipore-Sigma	G8540	3.42 mM
L-proline	Millipore-Sigma	81709	3.9 mM
L-glutamic acid	Millipore-Sigma	49449	1.7 mM
α-ketoglutaric acid	Millipore-Sigma	K1128	3.07 mM
Sodium hydroxide	Millipore-Sigma	S8045	10 N
D-glucose	Millipore-Sigma	G7021	100 mM
FBS (USDA approved; for tick medium)	Millipore-Sigma	F0926-500ML	10%
Bacto™ Tryptose Phosphate Broth	BD	260300	10%
Lipoprotein concentrate	MP Biomedicals	191476	0.10%
Normal rabbit serum	Pel-Freez	#31126-5	6.00%
Sodium bicarbonate	Millipore-Sigma	S6014	0.25%
HEPES	Millipore-Sigma	H4034	25 mM
CMRL1066 w/L-Glutamine (Powder)	US Biological	C5900	N/A
Sodium citrate tribasic dihydrate	Millipore-Sigma	S4641	2.38 mM
Yeastolate	BD	255772	2 g/L
Neopeptone	BD	211681	5 g/L
N-Acetyl-α-D-glucosamine	Millipore-Sigma	1079-25GM	N/A

Albumin, Bovine Fraction V	MP Biomedicals	160069	N/A
Rifampicin	Millipore-Sigma	557303	50 µg/ml
Phosphomycin	Millipore-Sigma	P5396	100 ug/ml
Amphotericin B	Gibco	15290-026	0.111111111
Sodium pyruvate	Millipore-Sigma	P5280	7.3 mM
RPMI-1640 Medium With L-Glutamine	Quality Biological	112-025-101	N/A
Fetal Bovine Serum (for HL-60s media)	Gemini Bio-Products	100-106	10 %
1X PBS	Quality Biological	114-058-101	N/A
Distilled water	Gibco	15-230-147	N/A
LB broth	Millipore-Sigma	L3022	N/A
LB agar with 100 ug/ml ampicillin	Quality Biological	50-751-7582	N/A
Ampicillin	Millipore-Sigma	A0166	100 µg/ml
**Reagents and Materials**			
Cellstar® cell culture flasks, 25 cm^2	Greiner bio-one	690-160	N/A
T-25 Vented Flasks	CytoOne	CC7682-4825	N/A
Cell culture plate with lid (6 well, flat bottom)	Millipore-Sigma	SIAL0516	N/A
iTaq™ Universal SYBR® Green Supermix	Biorad	1725121	N/A
Costar® cell culture plate with lid (24 well, flat bottom)	Corning	CLS3526-1EA	N/A
Mini-Protean® TGX™ gels	Biorad	456-9034	N/A
Trans-blot® Turbo™ Transfer pack, 0.2 µm PVDF	Biorad	1704156	N/A
Miller GP 0.2 µm filter unit	Millipore-Sigma	SLGP033RS	N/A
FALCON® 14 ml polypropylene round-bottom tube	Corning	352059	N/A
Nunc™ 96-Well polystyrene round bottom microwell plates	Thermo Scientific	262162	N/A
250 mm glass desiccator	Fisher Scientific	08-615B	N/A
1.5 ml microcentrifuge tubes	Thomas Scientific	1148T71	N/A

15 ml conical screw cap tubes	USA Scientific	5618-8261	N/A
50 ml conical screw cap tubes	USA Scientific	5622-7270	N/A
500 ml vacuum filter/storage bottle system, 0.2 µm	Corning	430773	N/A
27 gauge, 1/2" needle	BD	305109	N/A
White filter paper for CytoSep™ single funnel	Simport Scientific	M965FW	N/A
Richard-Allan Scientific™ three-step stain set	Thermo Scientific	3300	N/A
Epredia™ Cytospin™ 4 cytocentrifuge	Thermo Scientific	A78300003	N/A
Fisherbrand™ Superfrost™ plus microscope slides	Thermo Scientific	12-550-15	N/A
Halt™ phosphatase inhibitor cocktail (100x)	Thermo Scientific	78426	1:100
Halt™ protease inhibitor cocktail (100x)	Thermo Scientific	87786	1:100
10X RIPA	Millipore-Sigma	20-188	1X
Chloroform	Millipore-Sigma	288306	100%
TRIzol reagent	Ambion	15596018	N/A
Methanol anhydrous, 99.8%	Millipore-Sigma	322415-1L	100%
Ethyl alcohol, pure; 200 proof for molecular biology	Millipore-Sigma	E7023-1L	70-100%
HyClone™ water, molecular biology grade	Cytiva	SH3053801	N/A
Puromycin dihydrochloride	Millipore-Sigma	P9620	4 µg/ml
Pierce™ Anti-dykddddk affinity resin	Thermo Scientific	A36803	N/A
Pierce™ IP lysis buffer	Thermo Scientific	87787	N/A
2-β mercaptoethanol	Gibco	21985-023	5%
6X Laemmli buffer	Alfa Aesar	J60660	1X
Blocking grade blocker, non-fat skim milk	Biorad	1706404	5%
Bovine serum albumin	Millipore-Sigma	A2058	3%
Sodium dodecyl sulfate (SDS)	Millipore-Sigma	L6026	0.10%

Paraformaldehyde	Millipore-Sigma	P6148	4.00%
JumpStart™ REDTaq® ReadyMix™ Reaction Mix	Millipore Sigma	P1107	1X
Phusion® HF DNA Polymerase	New England BioLabs	M0530S	1 unit
**Commercial Assays**			
Pure Link RNA mini kit	Invitrogen	12183025	N/A
SF Cell Line 4D-Nucleofector™ X Kit L	Lonza Bioscience	V4XC-2012	N/A
Verso cDNA Synthesis Kit	Thermo Scientific	AB-1453B	N/A
Pierce BCA Protein Assay Kit	Thermo Scientific	23227	N/A
Pierce ECL Western Blotting Substrate	Thermo Scientific	32106	N/A
DNeasy® Blood and Tissue kit	Qiagen	69506	N/A
QIAamp® DNA Mini Kit	Qiagen	51304	N/A
QIAprep® Spin Miniprep Kit	Qiagen	27106	N/A
Mycoplasma testing kit	Southern Biotech	13100-01	N/A
Endofree Plasmid Maxi Kit	Qiagen	QGN-12362	N/A
Immobilon Forte Western HRP substrate	Millipore-Sigma	WBLUF0100	N/A
**Equipment**			
4D-Nucleofector™ System	Lonza Bioscience	AAF-1002	N/A
Cytospin 4	Thermo Scientific	A78300003	N/A
CFX96 Touch Real-Time PCR Detection System	Biorad	Discontinued	N/A
C1000 Touch Thermocycler	Biorad	1851148	N/A
**Cell Lines**			
I. scapularis ISE6 cells	Ulrike Munderloh, University of Minnesota		ISE6
**Organisms**			
One Shot Top10 Chemically Competent *E. coli*	Thermo Scientific	C404003	N/A
HL-60 cells	ATCC	CCL-240	NA
A. phagocytophilum HZ strain	Ulrike Munderloh, University of Minnesota		N/A

B. burgdoferi B31 clone MSK5	Jon Skare, Texas A&M University Health Science Center		N/A
**Plasmids**			
pAQUA-N1	Addgene	42888	N/A
pCMV-HA	Addgene	32530	N/A
pCMV-FLAG	Sino Biological, Inc	CV002	N/A
DsRed-N1	Addgene	54493	N/A
Donor vectors	Origene Technologies	custom	N/A
**Protein**			
Cas9-NLS-tagRFP protein	Genaxxon bioscience	S5306.0010	N/A

Supplementary Table 2: Primers used

Name	Type	Target Accession	Start position within mRNA or exon	Strand	Sequences (5'-3')
**pCMV-*xiap*-HA*¶**	Cloning	XM_002433822	1	F	GATTGCAG**GAATTC**ATGGTTGTCATCAGTATGGCG
			1014	R	GTATAGTT**GCGGCCGC**TCATGAAAGAAAAGCCTTAATGTTC
**pCMV-*p47*-FLAG*¶**	Cloning	XM_002433590	27	F	GGCC**GAATTC**ATGGCAGATTGTGCGGGGCG
			1175	R	GGCC**GTCGAC**TCACTTGATACGCTGGACGATAA
***xiap*-Dsred2-N1¶**	Cloning	XM_002433822	1	F	ACTCAGATCTC**GAGCTC**GATGGTTGTCATCAGTATGGCGATC
			1014	R	CGTCGACTGCA**GAATTC**CTGAAAGAAAAGCCTTAATGTTCTC
***p47*-AquaN1¶**	Cloning	XM_002433590	27	F	ATTCTGCA**GTCGAC**GGATGGCGGACTGCGAGGAAC
			1175	R	GCGACCGGT**GGATCC**CCTTTTATACGCTGCACAATG
p47	PCR	XM_002433590	NA	LHA-F	TGGTAGCTAAAGGCCTACAC
				RHA-R	GTCTTCACAAAGGGCAAAGG
xiap	PCR	XM_002433822	NA	LHA-F	TGGCGGTACCTTCCCAAATC
				RHA-R	AGTGTGCAACGGGATGCTAC
**donor cassette**	PCR	NA	NA	Puro-F	GCCTCTGTTCCACATACACTTC
				RFP-R	GATGCCCTGGGTGTGGTTGATG
***p47* sgRNA**	sgRNA	LOC120845803	498	F	CTGTACGGAGAACGACACAG

			468	R	AGCAGCGGTCATCGCGCAAC
***xiap* sgRNA**	sgRNA	LOC8050569	259	F	AAGGCGGGACTTTTTTACAA
			228	R	TCGGCGACGAGTACGTACAG
***A. phagocytophilum* 16S rRNA**	qRT-PCR	NC_007797	283	Ap16S_F	CAGCCACACTGGAACTGAGA
			400	Ap16S_R	CCCTAAGGCCTTCCTCACTC
I. scapularis p47	qRT-PCR	XM_040217354.2	93	p47_F	GCCAGGGCCAAGCTTTACC
			190	p47_R	CTTGGACGCTCCAGCGAC
I. scapularis kenny	qRT-PCR	XM_040500911.2	1169	Key_F	GCTCAGGACTTGGCAGGAAT
			1300	Key_R	CACCAGCTTGTCTTGGACCT
I. scapularis relish	qRT-PCR	XM_040501061.2	487	Relish_F	AGAATGTCCGCCACCGTTTTTTTCTGC
			595	Relish_R	CACGTGCACCGCCTCACCATGAAGG
I. scapularis actin	qRT-PCR	XM_029977298	896	I.s. actin_F	GGTCATCACAATCGGCAAC
			1003	I.s. actin_R	ATGGAGTTGTACGTGGTCTC

*Constructs used in McClure Carroll *et al.*, 2019

¶Bold letters are introduced restriction endonucleases

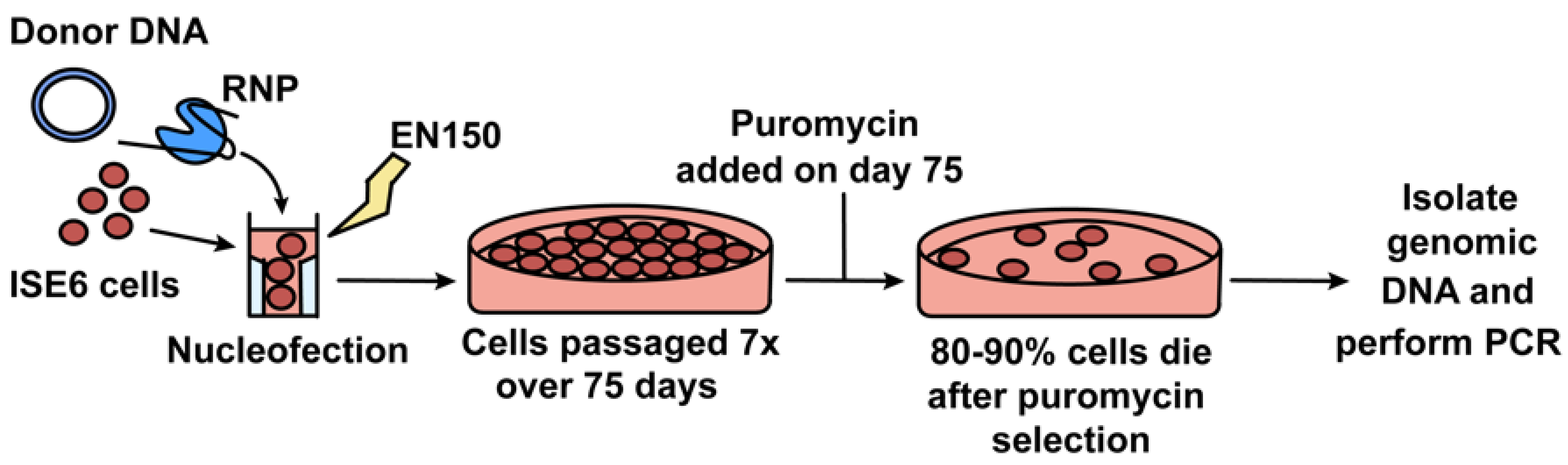

Singh *et al.* Figure S1

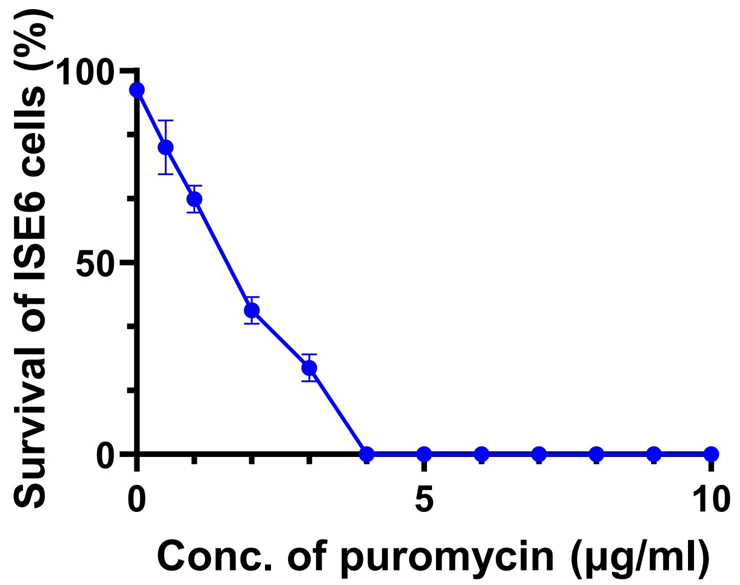

Singh *et al.* Figure S2

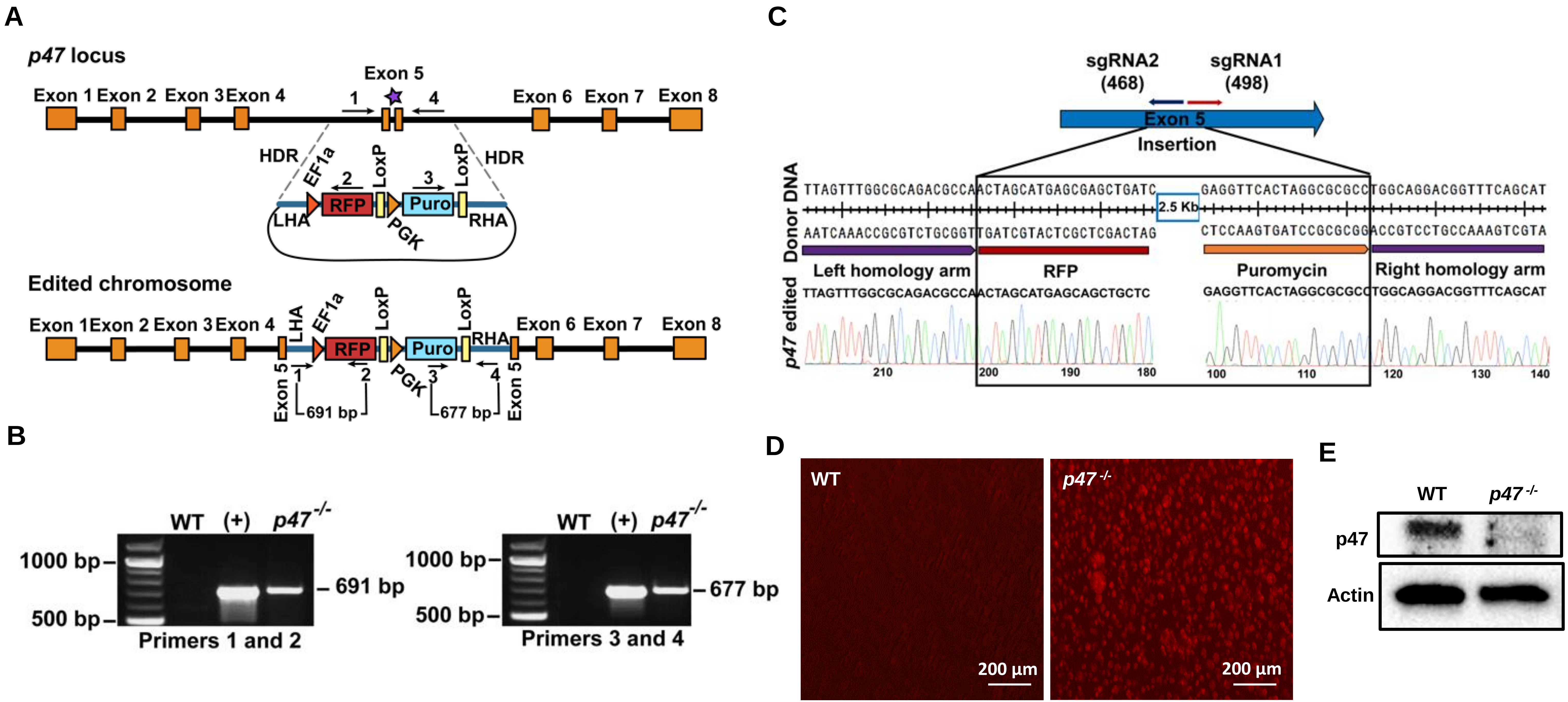

Singh *et al.* Figure S3

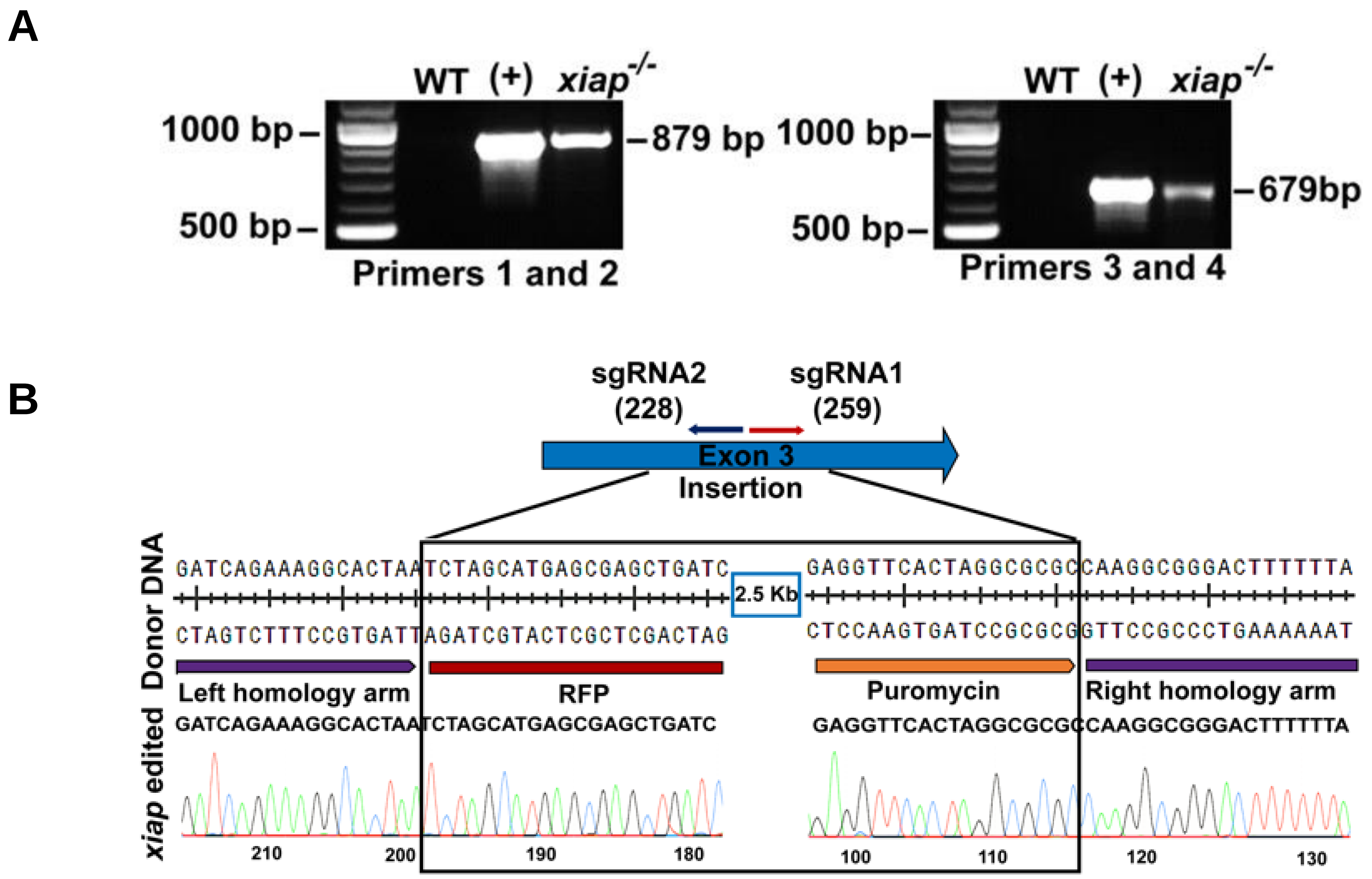

Singh *et al.* Figure S4

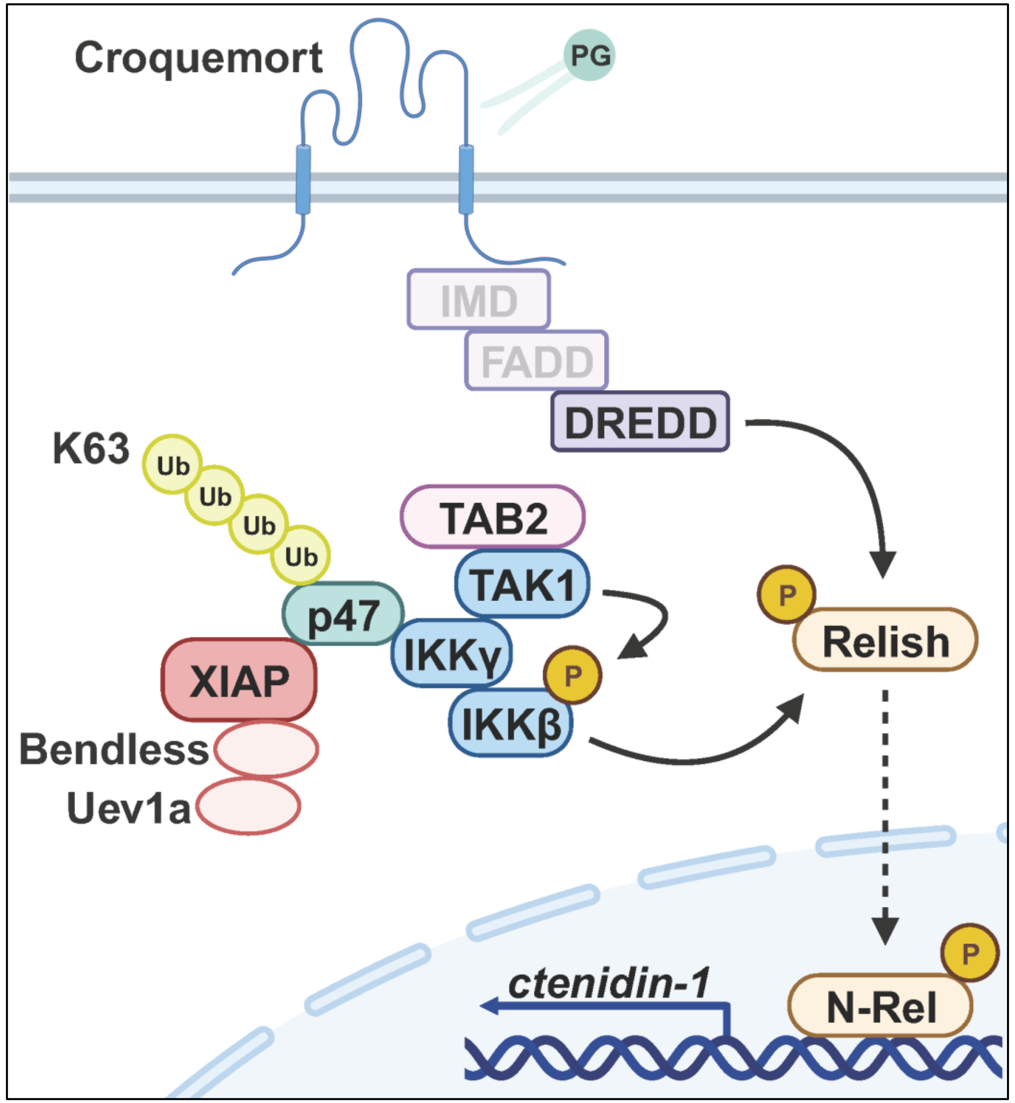

Singh *et al.* Figure S5
